# A Multidimensional Exploration Based on Hofstede’s Cultural Theory: An Empirical Study on Chinese Audience Acceptance of American Animated Films

**DOI:** 10.3390/bs15020164

**Published:** 2025-02-02

**Authors:** Tao Yu, Wei Yang, Ronghui Wu, Junping Xu, Jianhua Yang

**Affiliations:** 1 School of Communication, Qingdao University of Science and Technology, Laoshan Campus, No. 99 Songling Road, Qingdao 260061, China; 2 Department of Smart Experience Design, Kookmin University, Seoul 02707, Republic of Korea; ytkmu0517@kookmin.ac.kr (T.Y.); wytheyw@kookmin.ac.kr (W.Y.); wuronghui@kookmin.ac.kr (R.W.); xjp1110@kookmin.ac.kr (J.X.)

**Keywords:** Hofstede’s cultural dimensions, cross-cultural film consumption, cross-cultural acceptance, audience preferences, animated film acceptance, globalization and media

## Abstract

In the context of globalization, cross-cultural research is essential for understanding behaviors and values across different cultural backgrounds. The way audiences from diverse cultures interpret and accept film content significantly impacts the international dissemination and market performance of films. This study, grounded in Hofstede’s six cultural dimensions—power distance index (PDI), individualism vs. collectivism (IDV), uncertainty avoidance (UAI), masculinity vs. femininity (MAS), long-term vs. short-term orientation (LTO), and indulgence vs. restraint (IVR)—incorporates additional variables such as visual aesthetic appeal (VAA), narrative complexity (NCI), viewing motivation (VM), behavioral intentions (BIs), and brand loyalty (BL) to construct a multidimensional research framework. This framework aims to comprehensively examine the acceptance of American animated films among Chinese audiences and the cultural differences influencing such acceptance. Using structural equation modeling (SEM), this study analyzed the interrelationships between variables based on a sample of 507 participants with prior viewing experience. The findings reveal that different cultural dimensions significantly impact VM. PDI, UAI, and IDV exert significant negative influences on VM, with PDI being the most influential. Conversely, LTO and IVR do not demonstrate significant negative effects. In contrast, MAS, VAA, and NCI exhibit significant positive impacts on VM. Additionally, VM strongly influences audience acceptance, which, in turn, promotes the formation of BIs and repeated VM. This study extends the application of Hofstede’s cultural dimensions to the domain of cross-cultural media consumption, enriching the theoretical framework with additional dimensions and offering a novel perspective for cross-cultural research. Furthermore, the study uncovers the intricate interactions between cultural context and film content, proposing strategies to enhance the acceptance of cross-cultural films. These findings not only provide valuable insights for the production and marketing of animated films but also offer strategic guidance for filmmakers in diverse markets.

## 1. Introduction

Driven by the wave of globalization, cultural products have transcended geographical and cultural boundaries, becoming vital bridges connecting diverse nations and cultures (Jackson, 2004). The global circulation of cultural products fosters international economic collaboration and profoundly influences the cultural consumption patterns of global audiences (Choi & Park, 2014). Within this context, the film industry has demonstrated remarkable economic vitality, becoming a critical component of the cultural sector and a key medium for global cultural exchange (S. Chen & Zhang, 2023). According to data from Gower Street, from 2017 to 2023, global box office revenues exhibited a fluctuating trend—initial growth followed by a decline and gradual recovery. It is projected that global box office revenues will reach USD 34.5 billion in 2024 (Mitchell, 2023). As a significant branch of cinematic art, animated films, with their distinctive visual styles, boundless imagination, and broad audience appeal, have rapidly garnered a vast global following. Among them, American animated films, known for their innovative narratives and high-quality production standards, have long dominated the market (Barthel-Bouchier, 2012; Crane, 2014; Westcott, 2011). For instance, Disney’s Frozen achieved a global box office revenue of USD 1.28 billion, with overseas markets contributing USD 875 million, accounting for 68% of the total (Buck & Lee, 2014). Similarly, Pixar’s Incredibles 2 grossed USD 1.24 billion globally, with USD 634 million (51%) coming from international markets (Bird, 2018). Additionally, DreamWorks’ Kung Fu Panda 3 earned USD 154 million in China, making it one of the most successful American animated films in the Chinese market (Carloni & Nelson, 2016).

Although American animated films have achieved significant success in global markets, audiences from different countries and regions interpret and accept these films in diverse ways due to cultural differences (Cañas-Bajo et al., 2022; Craig et al., 2005; Fu, 2013). For example, the core theme of Finding Nemo in the United States centers on close family bonds and a father’s sense of responsibility, resonating deeply with American audiences (Brydon, 2009). In Japan, while the film is also popular, audiences tend to focus more on the emotional dependence and nuanced internal feelings between the characters (Lbata, 1981). Similarly, Pixar’s Coco achieved massive success in Mexico because of its faithful representation of the traditional Day of the Dead festival and its emphasis on family bonds, aligning with Mexico’s cultural reverence for family and ancestors (Crosthwait, 2020). However, in some Western countries, the film is often interpreted as a story of personal identity, emphasizing an individual’s connection to their past (Deszcz-Tryhubczak & Jaques, 2021). Additionally, DreamWorks’ Kung Fu Panda performed exceptionally well in the Chinese market not only because of its incorporation of Chinese cultural elements (W. Wang, 2017), but also due to its depiction of collectivism and teamwork values, which are familiar to Chinese audiences (Q. Wang, 2023). In contrast, American audiences are more likely to view the film as a story of self-discovery. These examples clearly illustrate that even the same film can be interpreted through distinct cultural lenses, with audiences drawing meaning from their own cultural value systems (Tipa, 2014). This raises several important questions: Why do these films achieve global success but are interpreted so differently across cultures (X. Wang et al., 2021; Zhou, 2016)? How do audiences from diverse cultural backgrounds interpret and accept foreign cultural products (Cañas-Bajo et al., 2022)? What accounts for the vastly different emotional responses to the same film among audiences from different cultures (Yamato, 2014)? How do they uniquely understand the values and social symbols embedded in these films (Crofts, 1992)? What do these cultural differences mean for the acceptance and commercial impact of films in global markets (Fu & Govindaraju, 2010)? Understanding these questions is crucial for exploring how cultural contexts shape audience responses to films, as well as for enhancing the cross-cultural communication capabilities of films.

The cultural values of Chinese audiences are deeply influenced by Confucian traditions, reflecting a tendency toward collectivism, significant power distance, and a pursuit of social harmony. In film acceptance behaviors, these values manifest as a preference for narratives emphasizing teamwork and family bonds, with weaker resonance for themes of individual heroism. Additionally, high uncertainty avoidance leads Chinese audiences to favor films with clear storylines and definite endings, while showing lower acceptance of open-ended narratives. At the same time, with socio-economic development, younger audiences increasingly value competition and a sense of achievement, while the importance of visual aesthetic appeal and narrative complexity reflects an elevated demand for aesthetic experiences. The diversity and evolution of these cultural values have profoundly influenced the acceptance of American animated films in the Chinese market.

To systematically explore the influence of cultural factors on Chinese audiences’ acceptance of American animated films, this study adopts Hofstede’s cultural dimensions theory. This framework encompasses six dimensions—power distance index (PDI), individualism vs. collectivism (IDV), uncertainty avoidance (UAI), masculinity vs. femininity (MAS), long-term vs. short-term orientation (LTO), and indulgence vs. restraint (IVR)—to understand how audiences’ values and behavioral tendencies differ across cultural backgrounds (Hofstede, 2011). These dimensions explain variations in cultural values, behavioral norms, and social interactions among global audiences, shedding light on how these differences shape their acceptance and interpretation of cultural content. However, film audiences’ experiences are not solely influenced by individual cultural dimensions (Dwyer, 2016). With globalization, the interplay between cultural backgrounds and individual perceptions or emotional experiences has become increasingly complex (Fu, 2013; Zhabskiy & Tarasov, 2023). For instance, visual aesthetic appeal (VAA) reflects varying cultural preferences for aesthetic styles, while narrative complexity (NCI) interacts with long-term or short-term orientation and uncertainty avoidance, shaping audiences’ tolerance and expectations for complex plots (R. Chen et al., 2010; Masuda et al., 2008). The Individualism vs. collectivism dimension further reveals audiences’ differing needs for individualized expression versus collective values. These variables interact dynamically, contributing to a multidimensional impact on film acceptance. To address these complexities, this study introduces additional variables, including visual aesthetic appeal (Wingstedt et al., 2010), narrative structure (Huang & Grizzard, 2022), viewing motivation (VM) (Yoo et al., 2020), acceptance (AC) (Quan & Rui, 2023), behavioral intentions (BIs) (X. Lu & Lo, 2007), and brand loyalty (BL) (Picón-Berjoyo et al., 2016). These newly incorporated variables enrich the theoretical framework by constructing a more comprehensive analytical model from multiple levels and perspectives. This framework seeks to explore how these factors collectively influence audiences’ acceptance and reactions to cross-cultural film content. By employing a multidimensional approach, this study aims to reveal how cultural and individual experiential factors jointly shape audience acceptance of films, offering nuanced insights and support for the cross-cultural dissemination of animated films.

Based on the above context, this study aims to investigate, through empirical research, how the cultural values of Chinese audiences influence their acceptance of American animated films. Specifically, this study seeks to answer the following questions:

(1) How do the cultural values of Chinese audiences specifically affect their acceptance behavior toward American animated films?
(2) Which cultural dimensions play a critical role in shaping audience preferences and motivations for animated films?
(3) How can the findings provide strategic recommendations for the development of American animated films in the Chinese market?

The primary contributions of this research are as follows: Theoretical Contribution: This study innovatively applies Hofstede’s cultural dimensions theory to cross-cultural acceptance research in animated films, addressing a gap in the field and expanding the application scope of Hofstede’s framework. Empirical Contribution: Through an analysis of data from Chinese audiences, the study reveals how cultural dimensions significantly influence acceptance behaviors, enriching the understanding of cross-cultural differences in film consumption behavior. Cross-cultural Exchange Contribution: By examining how Chinese cultural backgrounds affect the acceptance of American animated films, this study provides practical recommendations for cultural adaptation strategies for filmmakers in the Chinese market, thereby promoting global cultural exchange and inclusivity.

**Figure 1:**
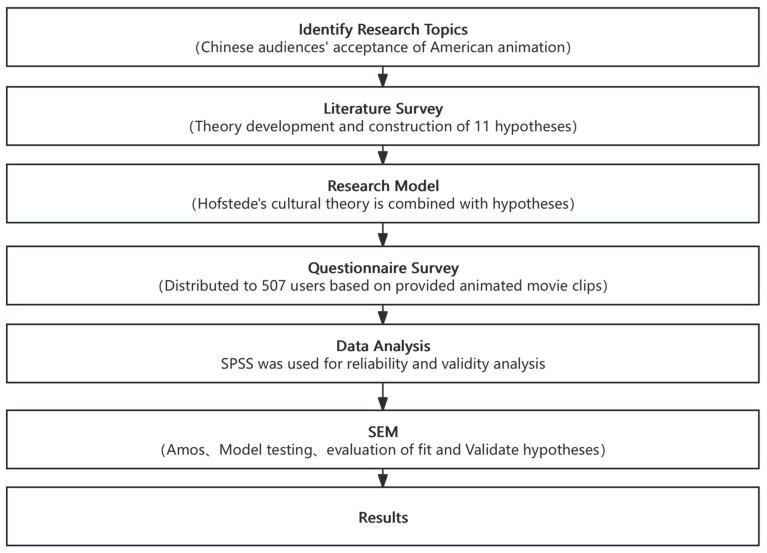
Flowchart of the study.

The structure of this paper is as follows: Section 2 reviews the current state of research on the cross-cultural acceptance of animated films and Hofstede’s cultural dimensions theory. Section 3 presents the research model and hypotheses. Section 4, Section 5, Section 6 and Section 7 cover model evaluation, discussion, conclusions, limitations, and directions for future research. Figure 1 illustrates the methodological workflow of this study.

## 2. Literature Review

### 2.1. Research Status of Cross-Cultural Film Acceptance

In the context of globalization, scholars have explored how different cultural backgrounds shape audience interpretations and evaluations of films through cross-cultural film reception studies. Existing research primarily focuses on the collision and integration of Western and non-Western cultures (Cooper-Chen, 2012). For instance, Chu examined the reception of Chinese films in the West, highlighting how cultural and political contexts profoundly influence audience evaluations of foreign films, thereby reflecting the complexities of cross-cultural communication (Chu, 2014). Chen and Liu, through their study of Disney’s Turning Red, demonstrated how emotional resonance facilitates the acceptance of hybrid cultural animated content by Chinese audiences (R. Chen & Liu, 2023). Giovanni analyzed depictions of foreign cultures in Disney animations, revealing how Western cultural values influence cinematic cultural symbols and their reception among non-Western audiences (Di Giovanni, 2003). Hernández and Hirai investigated the impact of historical, social, and cultural factors on audience perceptions (Hernandez & Hirai, 2015). Similarly, Tipa explored the unique role of animation in cross-cultural communication (Tipa, 2014). Further studies, such as those by Oliva, compared how Disney and Studio Ghibli address themes like environmental conservation and gender roles, showcasing differences in cultural acceptance of animated films between Eastern and Western audiences (Monleón Oliva, 2020).

Chinese audiences are deeply influenced by core cultural values such as collectivism, respect for hierarchical structures, and an emphasis on social harmony. These values shape their media consumption behaviors and their acceptance of foreign cultural products. For example, collectivism encourages appreciation for narratives that highlight family bonds and team spirit, while high respect for authority may lead to skepticism toward stories that challenge hierarchical structures. In the Chinese context, with the rapid growth of the film market, the reception and interpretation of films from different countries by Chinese audiences have increasingly become a focus of attention. This is particularly evident when audiences encounter content from Western countries, where cultural differences become more pronounced. Tingting and Fan compared the representation of Chinese cultural elements in Mulan and Kung Fu Panda, analyzing the impact of cultural differences on audience acceptance (Tingting & Fan, 2016). Meng and Yu examined the motivations and gratifications of Chinese audiences watching Japanese animation, highlighting the role of cultural proximity and familiarity in shaping viewing motivations (Meng & Yu, 2019). Ishii further analyzed the relationship between Chinese audiences’ preferences for Japanese animation and patriotism, clarifying how factors such as age and internet usage influence acceptance (Ishii, 2013). These studies explore how films are received in different cultural contexts, particularly how audiences evaluate content based on their own cultural values and expectations. Overall, films may elicit vastly different evaluations across cultural environments—a movie considered a classic in one culture may be constrained by misalignment with values in another. Elements such as language, customs, and behaviors embedded in a film significantly influence its effectiveness in cross-cultural dissemination, offering key insights for future research directions.

Academic research has also delved into how cultural differences impact the popularity and market performance of films. Studies suggest that the cross-cultural success of films depends not only on their artistic value and entertainment but also on their ability to evoke emotional resonance across diverse cultures (Budeva, 2010). To achieve this, international filmmakers increasingly emphasize incorporating cross-cultural elements to meet the needs of global audiences (Manning & Shackford-Bradley, 2010). This can involve integrating universally accepted themes, such as friendship, family, and courage, designing characters with broad appeal, or adapting region-specific details to align with the aesthetic expectations of various cultural groups (Brown, 2021). This approach not only enhances a film’s global appeal but also mitigates the risk of perpetuating cultural stereotypes or misunderstandings (Di Giovanni, 2003). At the same time, such careful optimization fosters deeper emotional resonance, enabling the story to connect with audiences from diverse cultural backgrounds (Crane, 2014). Furthermore, film criticism and theoretical analysis have highlighted the role of “cultural legitimacy” in evaluating films (Yaren & Karademir Hazır, 2020). Researchers argue that whether a film is recognized as a “legitimate” cultural expression across different cultures often hinges on political and ideological influences (Verboord, 2014). Thus, as a vital medium for cross-cultural dialogue, films exert influence far beyond entertainment, profoundly shaping cultural identity and social interactions.

In conclusion, research on cross-cultural film reception offers critical insights into understanding the dynamics of globalization through an analysis of audience reception patterns across various cultural contexts. These studies not only highlight the role of films in fostering global cultural exchange but also provide valuable guidance for filmmakers and distributors in developing effective production and distribution strategies. By addressing these issues in depth, film scholars and creators can better understand and harness the potential of films to shape cross-cultural identity and mutual understanding. For the convenience of researchers, Table 1 summarizes key studies and discussions on animated films across different dimensions.

### 2.2. Hofstede’s Cultural Dimensions Theory

Hofstede’s cultural dimensions theory identifies key differences among cultures in terms of power structures, individualism versus collectivism, uncertainty management, gender role perceptions, and perspectives on time and self-control. This framework has been widely adopted across academic research and practical applications.

This theory provides a framework for understanding and predicting individual behavior across diverse cultural contexts, especially in the domain of cross-cultural communication, where it is used to analyze communication barriers and adaptation strategies (Minkov & Kaasa, 2021). For instance, Huang’s research highlights that power distance and collectivism significantly impact communication efficiency in cross-cultural management within Northeast Asian enterprises (Huang et al., 2021). In the field of information technology, Gaspay et al. demonstrated how Hofstede’s dimensions aid in understanding cross-cultural teams’ attitudes toward technology, offering theoretical support for international collaboration (Gaspay et al., 2009). Additionally, Beugelsdijk and Welzel integrated Hofstede’s dimensions with Inglehart’s cultural dynamics theory to analyze cross-national cultural differences, underlining its explanatory power in understanding cultural transitions (Beugelsdijk & Welzel, 2018). In social psychology, Gouveia and Ro compared Hofstede’s model with Schwartz’s cultural framework, illustrating the effectiveness of individualism and collectivism in explaining social and economic variables, further emphasizing its value in social psychology (Gouveia & Ros, 2000). The education sector also benefits from this theory; Gao’s research revealed that adapting to cultural differences in teaching materials significantly enhances students’ learning outcomes (Yan, 2021). In the domain of animated films, current research remains limited. For example, Dong analyzed audience reviews of The Wandering Earth 2 in China and the U.S., uncovering the impact of power distance and long-term orientation on acceptance (Dong, 2023). Çakmak’s study of the Turkish animation Rafadan Tayfa demonstrated how cultural dimensions like collectivism enhance adaptability across diverse platforms (Yılmaz et al., 2019). Favaretto et al. emphasized that cultural symbols in visual content effectively reveal cultural differences (Favaretto et al., 2016). Overall, the acceptance of animated films remains an underexplored field. Despite their international appeal through unique visual styles and educational entertainment, the cultural values and acceptance mechanisms within the cross-cultural dissemination of animated films have not been thoroughly examined. Unlike live-action films, animated films often employ cultural symbols and character representations that are more symbolic and universal. Thus, utilizing Hofstede’s framework to investigate the acceptance of animated films across cultural contexts holds significant academic value and practical potential. This approach can guide animation production and global market strategies with more targeted insights.

## 3. Theoretical Framework and Hypotheses

This study adopts Hofstede’s cultural dimensions theory as its analytical framework, integrating six core dimensions, PDI, IDV, UAI, MAS, LTO, and IVR, to explore how these dimensions influence Chinese audiences’ acceptance of American animated films. Additionally, the study incorporates VAA, NCI, VM, and AC to complement Hofstede’s framework, addressing the limitations of traditional cultural dimensions theories in capturing audience experiences. These variables focus on the sensory and cognitive appeal of film content, working synergistically with the cultural influences emphasized by Hofstede’s dimensions. Furthermore, the term masculinity vs. femininity has recently been updated to Motivation Towards Achievement and Success. This renaming reflects a modern perspective and responds to concerns regarding binary gender interpretations. However, this study retains the original terminology, masculinity vs. femininity, for several reasons: first, to maintain academic consistency and theoretical integrity, aligning with Hofstede’s original framework, which has been extensively validated in existing literature; second, because the traditional definition of MAS—encompassing preferences for achievement, heroism, confidence, and material success—remains significant in capturing the evolving cultural dynamics in China, such as the increasing emphasis on competition and personal achievement; and third, to ensure methodological consistency, as the study’s data collection and variable design were based on Hofstede’s original definitions. Retaining the original terminology avoids potential confusion and ensures clarity and coherence in the research.

Specifically, VAA aligns with IDV; for instance, individualistic cultures often prefer unique designs, while collectivistic cultures emphasize harmonious expressions. NCI corresponds closely with UAI, as complex narratives tend to appeal more to low-UAI cultures, whereas high-UAI cultures favor clearly structured films. VM and AC reflect the interplay between cultural values and personal preferences, jointly influencing viewing choices and BL. By integrating these variables, this study extends the application of Hofstede’s cultural dimensions theory, offering a more comprehensive framework for understanding cross-cultural film acceptance behavior.

### 3.1. Hofstede’s Six Cultural Dimensions

#### 3.1.1. Power Distance Index (PDI)

PDI refers to the degree to which members of a society accept unequal power distribution. In high power distance cultures, such as China, hierarchical structures and authority are more widely acknowledged (J. Lu et al., 2019). Lei found that in high power distance cultures, audiences are more likely to identify with authoritative characters and resonate less with anti-authority themes (Lei et al., 2021). This contrasts with American animated films, which often feature narratives of defying authority and breaking hierarchical norms, potentially causing cultural conflict in a Chinese context (X. Wang et al., 2021).

Thus, we propose the following hypothesis:

**H1:**  *PDI negatively affects Chinese audiences’ motivation to watch American animated films.*

#### 3.1.2. Individualism vs. Collectivism (IDV)

The IDV dimension highlights differing cultural perspectives on the relationship between individuals and groups. China represents a prototypical collectivist culture, emphasizing family, group cohesion, and social responsibility (G. Wang & Chen, 2010). In film narratives, themes centered on individual heroism and independence may conflict with Chinese audiences’ preference for values like teamwork and family unity (S. Chen, 2022).

Thus, we propose the following hypothesis:

**H2:**  *IDV negatively impacts Chinese audiences’ motivation to watch American animated films.*

#### 3.1.3. Uncertainty Avoidance (UAI)

UAI reflects a society’s tolerance for uncertainty and ambiguity. China, characterized as a moderate to high uncertainty avoidance culture, often favors clear rules and structures to reduce uncertainty (Feng, 2011). Consequently, open-ended or ambiguous narratives common in American animated films may not align with Chinese aesthetic preferences (He, 2020), as they tend to favor films with explicit moral guidance and definitive conclusions (Jia, 2009).

Thus, we propose the following hypothesis:

**H3:**  *Uncertainty avoidance negatively impacts Chinese audiences’ motivation to watch American animated films.*

#### 3.1.4. Masculinity vs. Femininity (MAS)

The MAS dimension describes a society’s varying emphasis on values such as competition and achievement (masculinity) versus harmony and care (femininity) (Geng Song, 2010). Chinese culture demonstrates a balance in this dimension, valuing both achievement and success (masculinity) and harmony and care (femininity) (Zhang, 2019). This suggests that films heavily emphasizing competition and individual achievement may not resonate as strongly in the Chinese market as they do in Western markets. Conversely, films highlighting family harmony and emotional care are more likely to elicit emotional resonance among Chinese audiences (He, 2020).

Thus, we propose the following hypothesis:

**H4:**  *MAS positively influences Chinese audiences’ motivation to watch American animated films.*

#### 3.1.5. Long-Term vs. Short-Term Orientation (LTO)

The LTO dimension reflects a society’s attitudes toward future planning and traditions. China is characterized by a long-term oriented culture, emphasizing future achievements and sustained effort (Dong, 2023). As a result, the themes of short-term adventures and instant gratification commonly found in American animated films may not fully align with Chinese cultural values. In contrast, characters and storylines that embody patience and persistent effort are more likely to be accepted and appreciated by Chinese audiences (Hofstede & Minkov, 2010).

Thus, we propose the following hypothesis:

**H5:**  *LTO negatively influences Chinese audiences’ motivation to watch American animated films.*

#### 3.1.6. Indulgence vs. Restraint (IVR)

The dimension of IVR reflects a society’s attitude toward desires and enjoyment. Chinese culture leans toward restraint, emphasizing self-discipline and control over desires (Crittenden, 2016). This suggests that excessive depictions of hedonism and indulgence in films might conflict with the values of Chinese audiences, who are more inclined to appreciate characters and storylines that exhibit self-restraint and a sense of responsibility (Jia, 2009).

Therefore, we propose the following hypothesis:

**H6:**  *IVR negatively affects Chinese audiences’ motivation to watch American animated films.*

### 3.2. Visual Aesthetic Appeal (VAA) and Narrative Complexity (NCI)

#### 3.2.1. Visual Aesthetic Appeal (VAA)

VAA is not only one of the core factors in attracting audiences but also directly influences their emotional responses and attitudes toward film content. Chen noted that the visual effects in animated films often enhance the viewing experience through color (H. Sun, 2011), animation fluidity, and compositional creativity, though the acceptance of these visual elements may vary across cultural backgrounds (Kostoulas et al., 2017). Given that the visual aesthetic preferences of Chinese audiences are influenced by their cultural traditions, VAA as a dimension can effectively explain the success or challenges faced by American animated films in the Chinese market.

Therefore, we propose the following hypothesis:

**H7:**  *VAA positively influences Chinese audiences’ motivation to watch American animated films.*

#### 3.2.2. Narrative Complexity (NCI)

NCI, such as multiple storylines, non-linear storytelling, and multi-layered character development, can engage audiences cognitively and enhance the rewatch value of films (X. Chen, 2018). However, the acceptance of narrative structure varies significantly across cultural backgrounds (J. Lu et al., 2019). While complex narratives may achieve success in Western markets, they could pose comprehension challenges for some Chinese audiences, who tend to prefer linear storytelling with clear moral guidance (Wu, 2023). Introducing NCI as a dimension helps to explore cultural differences in how Chinese audiences interpret the plot of American animated films (Busselle & Bilandzic, 2009).

Therefore, we propose the following hypothesis:

**H8:**  *NCI positively influences Chinese audiences’ motivation to watch American animated films.*

### 3.3. Viewing Motivation (VM), Acceptance (AC), Behavioral Intentions (BIs), Brand Loyalty (BL)

In cross-cultural film consumption, VM, AC, BIs, and BL are key factors in understanding audience behavior (Du et al., 2022; J. Lu et al., 2019). These factors not only influence audience choices and viewing experiences but also determine their long-term support for film brands. Research indicates that these factors are influenced both by film content and style, as well as cultural background. When analyzing Chinese audiences’ acceptance of American animated films, these three aspects help reveal the characteristics of their cross-cultural consumption behavior (See Kam Tan, 2011). VM, as the driving force behind audience film choices, is shaped by psychological needs and cultural preferences (X. Lu & Lo, 2007). BIs, the audience’s inclination to act after viewing, include tendencies to recommend, rewatch, or purchase related products, often influenced by both viewing motivation and acceptance (Danaher & Dagger, 2012). BL refers to the enduring preference and support audiences show towards a specific film brand, which in turn reinforces future viewing motivation, creating a positive cycle (Jacoby & Kyner, 1973).

Therefore, we propose the following hypothesis:

**H9:**  *VM positively influences Chinese audiences’ acceptance of American animated films.*

**H10:**  *Chinese audiences’ acceptance of American animated films positively influences their BIs, including the tendency to recommend and rewatch.*

**H11:**  *Chinese audiences’ acceptance of American animated films positively influences their BL.*

### 3.4. Proposed Research Model

**Figure 2:**
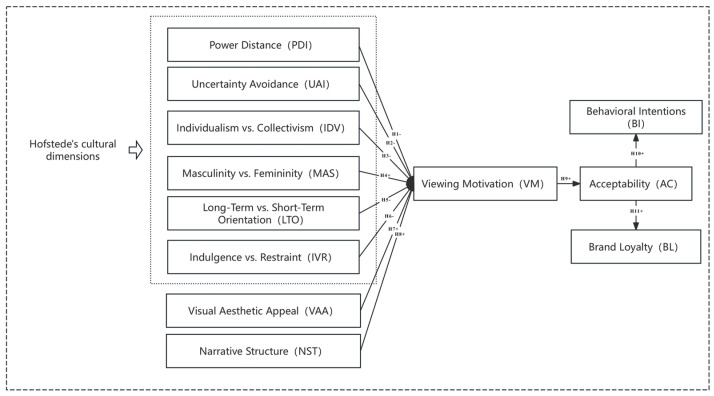
Research hypothesis model.

Hofstede’s cultural dimensions, serving as inputs, reflect the values and behavioral tendencies of audiences from different cultural backgrounds. Building on previous research that identified visual aesthetic appeal and narrative structure as critical factors in cross-cultural film acceptance and evaluation, these two cultural dimensions were additionally incorporated into the model. Combining Hofstede’s six cultural dimensions with visual aesthetic appeal and narrative complexity, the model explores four key factors in film consumption behavior. This study aims to reveal how cultural factors influence viewing motivation and acceptance, which in turn affect audience behavior and brand loyalty. This comprehensive perspective provides insights for understanding and predicting film consumption behaviors across diverse cultural backgrounds (Figure 2).

## 4. Research Methodology

### 4.1. Questionnaire Development

The questionnaire data collection consisted of two parts. The first part gathered participants’ basic demographic information, such as gender, age, education level, and animated film viewing habits. The second part aligned with the research model and included 12 variables, all designed based on prior literature to ensure the validity and reliability of the measurement indicators. To further improve the quality of the questionnaire, four experts in the field of animated films were invited to review the items. Following in-depth discussions, an initial set of 48 core items was identified. Subsequently, through statistical analysis and validation, the number of items was refined to 44, which were incorporated into the final measurement tool.

**Figure 3:**
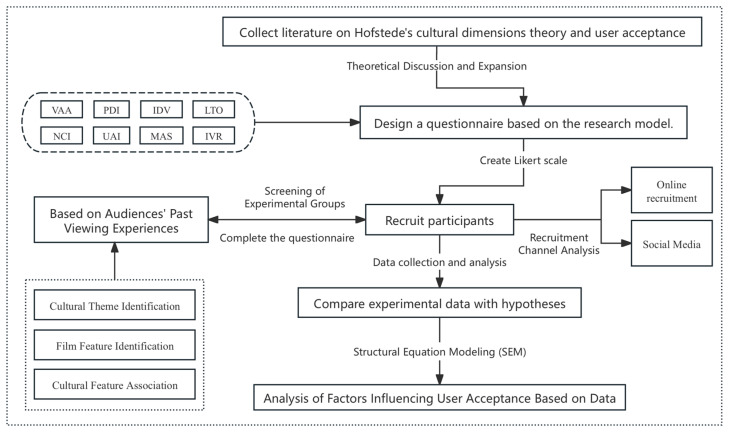
Experimental research process diagram.

The questionnaire employed a five-point Likert scale (1 = strongly disagree, 5 = strongly agree), a widely recognized method for effectively measuring the intensity and direction of respondents’ attitudes. Since the original questionnaire was in English and the study was conducted in China, three professional English translation experts were engaged to perform multiple rounds of translation and proofreading to minimize translation errors and semantic ambiguities. After completing the design, a small-scale pilot test was conducted involving 30 participants who had watched American animated films. The aim was to evaluate the questionnaire’s logical coherence and presentation. Based on feedback from researchers and participants, the structure and phrasing of the questionnaire were optimized. Notably, the 30 pilot test participants were excluded from the formal analysis to prevent potential biases caused by repeated exposure to the questionnaire (Figure 3).

All participants signed an informed consent form prior to completing the questionnaire to ensure their rights were protected. Participants were informed that the data would be used solely for academic research and that their personal privacy would be strictly safeguarded. The questionnaire items and the reference list are provided in Table 2.

### 4.2. Data Collection and Participant Statistics

This study adopts a cross-sectional online survey design, with data collected through the Chinese professional online survey platform Wenjuanxing. The data collection utilized a snowball sampling technique across social media platforms, with participants having the chance to win rewards through a lottery, including (1) a CNY 5 WeChat red envelope, (2) a CNY 10 WeChat red envelope, and (3) a certificate of participation and a thank-you letter. To mitigate the limitations of snowball sampling, the questionnaire was initially disseminated through WeChat and QQ groups, avoiding complete reliance on personal recommendations, and respondents were encouraged to invite individuals from diverse social backgrounds to participate, enhancing sample diversity and reducing bias toward specific groups.

Additionally, to further explore Chinese audiences’ acceptance of American animated films, this study collaborated with animation industry experts to select 15 animated film clips (Table 3) from a wide range of films, covering all the required research variables. If the provided film clips did not fully capture relevant content, participants were allowed to add additional clips.

Screening Criteria: Film Type and Style: The selected films cover a variety of styles and types of animated films, including classic Disney productions, Pixar animations, and films produced by DreamWorks. These films have a broad global influence and are highly recognized, ranging from family-friendly films to those with complex narrative structures. Global Influence: The selected films have significant box office revenue and cultural influence worldwide. For example, works such as *Frozen* and *Zootopia* not only succeeded in the United States but also sparked widespread discussions and received high praise in multiple countries and regions. Cultural Diversity: To ensure a thorough analysis of film acceptance across different cultural backgrounds, the selected films include various cultural symbols and social themes, such as Mexican traditions in Coco, Chinese culture in Kung Fu Panda, and family themes in Finding Nemo. Story Content and Character Traits: The selected films include those with rich narratives and distinct character traits, effectively representing cultural dimensions such as individual heroism, family bonds, teamwork, and moral conflicts, which align with the research focus on the relationship between audience cultural backgrounds and film content. Influence and Representativeness: All selected films are highly representative and influential, showcasing the acceptance of different cultures from multiple dimensions, such as visual effects, narrative complexity, and character design. These films have a significant impact on global markets and resonate deeply with audiences in various countries and regions. Audience Acceptance and Accessibility: The selected films have a broad audience base and are readily accessible for related data collection, ensuring the research can effectively measure the acceptance and influence of films across different cultural backgrounds. Through these films, we can compare Chinese audiences’ interpretations and acceptance of the film content with those of audiences from other countries.

Finally, a questionnaire with responses to more than 10 clips was defined as a valid response. A total of 650 questionnaires were distributed, 590 were returned, and 507 were deemed valid for the study.

The demographic analysis reveals a balanced gender distribution among respondents, with males comprising 48.7% and females 51.3%. In terms of age, the 18–25 age group represents the highest proportion at 36.9%, followed by the 26–30 age group at 25%, together accounting for 61.9% of the sample. Regarding educational attainment, 73.8% of respondents have a higher education degree (Table 4).

## 5. Results

This study conducted multiple data validation steps, including common method bias testing, reliability analysis, validity analysis, and correlation testing. The results demonstrated high reliability and validity of the data, with significant correlations among the dimensions. Finally, the relationships between dimensions and acceptance were verified using structural equation modeling, ensuring the reliability of the conclusions. These analyses provided robust scientific support for the study.

As shown in Table 5, the response and popularity rates are noticeably higher for the following American animated films: Finding Nemo (2003), Despicable Me (2017), and Zootopia (2016).

As shown in Table 6, since all data were collected through questionnaire surveys, there may be a risk of common method bias due to the uniform data collection method, which can result in systematic directional error in the data (Podsakoff et al., 2003). The Harman single-factor test is typically used to examine common method bias by including all Likert scale items in a factor analysis. If the variance explained by the first factor exceeds 40%, common method bias is likely present; if it is below 40%, common method bias is either absent or not severe.

In this survey, the first factor explained 32.249% of the variance, which is below the 40% threshold, indicating that common method bias is not significant in this study.

As shown in Table 7, the reliability coefficients for the 12 variables in this survey are 0.858, 0.859, 0.863, 0.877, 0.866, 0.866, 0.878, 0.857, 0.861, 0.879, 0.865, and 0.862, all exceeding 0.8. Therefore, the data results are generally stable and exhibit high reliability. This indicates that the questionnaire overall demonstrates good reliability, with a certain level of stability and dependability (Taherdoost, 2016).

As shown in Table 8 below, the approximate chi-square is 13,861.344, with a degree of freedom of 1128 and a *p*-value of 0.000. The KMO value is 0.944, which is greater than 0.9, indicating that the data are suitable for factor analysis.

The calculation results are presented in Table 9. From the data, it can be observed that the fit indices meet the analysis standards: χ^2^/df = 1.046 < 3, RMSEA = 0.01 < 0.05, SRMR = 0.036 < 0.05, NFI = 0.926 > 0.9, CFI = 0.996 > 0.9, and GFI = 0.921 > 0.9. In summary, all fit indices for the variables in the questionnaire data meet the required standards, indicating a good model fit and high overall suitability. The questionnaire demonstrates strong construct validity (Rigdon, 1996).

Based on the descriptive statistics and the reliability and validity test results, a structural equation model was established using AMOS 23.0 software. Variables included PDI, UAI, IDV, MAS, LTO, IVR, VAA, NCI, VM, AC, BIs, and BL to examine the comprehensive relationships among variables and to organize the results of all hypotheses.

**Figure 4:**
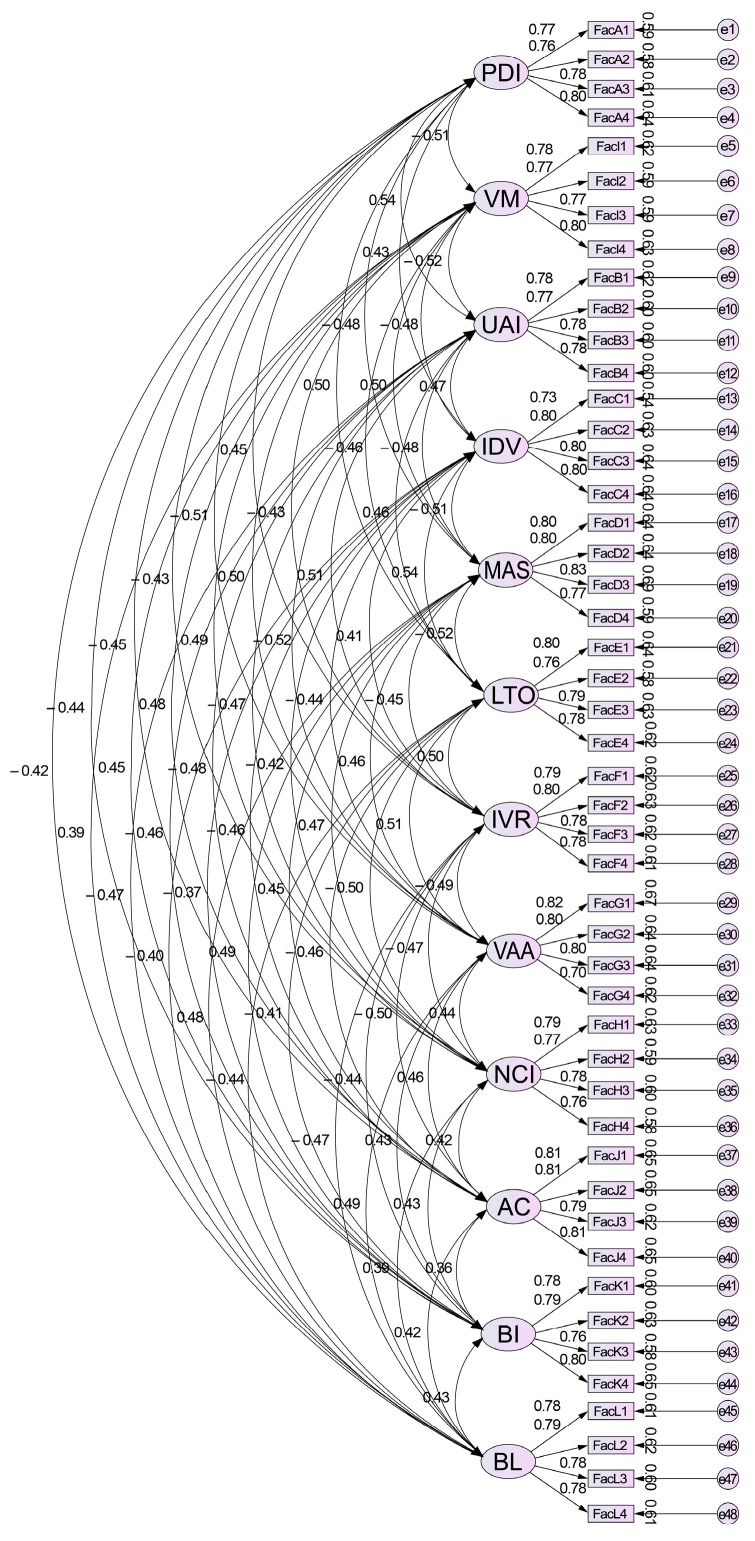
Standardized output confirmatory factor analysis model diagram.

The confirmatory factor analysis model, as shown in Figure 4, was established using AMOS 23.0 software to examine the overall fit of the variables.

As shown in Table 10, the correlation coefficients between each pair of variables do not exceed the values on the diagonal for the corresponding variables, indicating good discriminant validity for the model (Hair et al., 2012).

**Figure 5:**
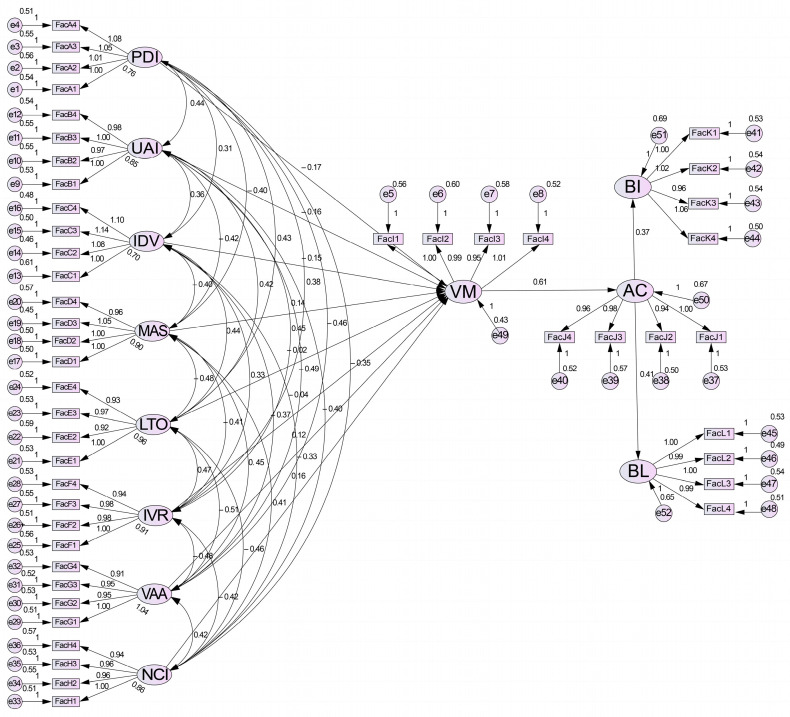
Initial structural equation model diagram.

As shown in Figure 5, based on the descriptive statistics and the reliability and validity test results, a structural equation model was established using AMOS 23.0 software. Variables included PDI, UAI, IDV, MAS, LTO, IVR, VAA, NCI, VM, AC, BIs, and BL to examine the comprehensive relationships among variables and to organize the results of all hypotheses.

The purpose of the fit indices is to verify the degree of alignment between the hypothesized model and the data. After constructing the structural equation model, a model fit test was conducted. The reference values for model fit indices are shown in Table 11.

As shown in Table 12, the model fit results for this study are as follows: χ^2^ = 1383.481, χ^2^/DF = 1.329 (meeting the standard of being less than 3), GFI = 0.903, AGFI = 0.89 (both greater than 0.8), RMSEA = 0.025 (less than 0.05), NFI = 0.904, and CFI = 0.974 (both greater than 0.9). All model indices meet the testing standards, indicating good model fit (Gerbing & Anderson, 1992).

**Figure 6:**
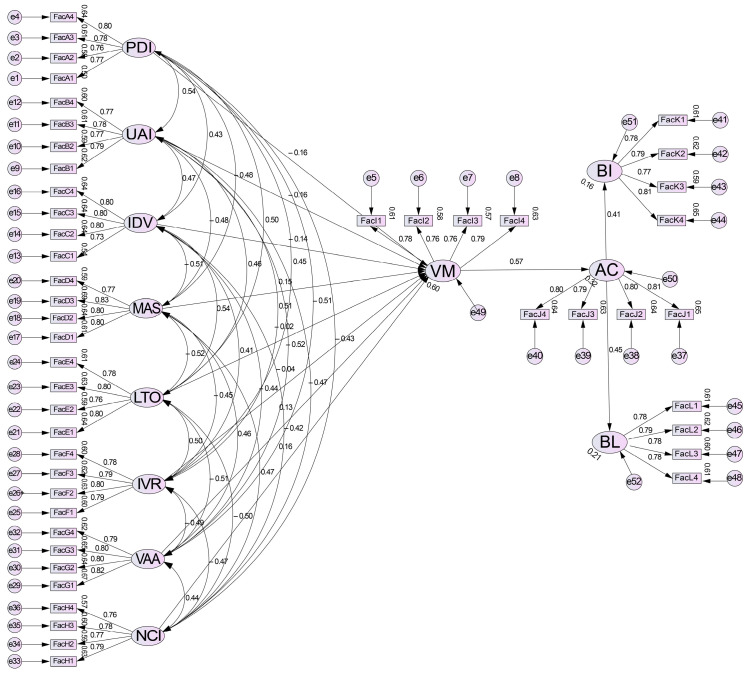
Standardized estimated structural equation model diagram.

As shown in Figure 6 and Table 13, all model indices meet the testing standards, indicating good model fit (Gerbing & Anderson, 1992).

According to the analysis results, PDI, UAI, and IDV have a significant negative effect on VM, while MAS, VAA, and NCI have a significant positive effect on VM. The effects of LTO and IVR on VM are not significant. Additionally, VM has a significant positive effect on AC, and AC has a significant positive effect on both BIs and BL. Therefore, hypotheses H1, H2, H3, H4, H7, H8, H9, H10, and H11 are supported, while H5 and H6 are not supported.

## 6. Discussion

### 6.1. Main Findings

The results indicate that Hofstede’s cultural dimensions of PDI, UAI, and IDV exert a significant negative impact on the viewing motivations of Chinese audiences. This finding suggests that in cultural contexts characterized by high power distance and uncertainty avoidance, audiences tend to prefer content aligned with their cultural values, while the themes of individual heroism and narrative styles prevalent in American animated films may conflict with their preferences. These findings align with studies by Craig and Lee, which highlight the significant influence of cultural values on audience acceptance of foreign cultural content in cross-cultural communication (Craig et al., 2005; F. L. F. Lee, 2008; Zhou, 2016). Moreover, Budeva emphasized in her research that in high power distance cultures, audiences exhibit higher acceptance of content emphasizing authority and harmony, further supporting the conclusions of this study (Budeva, 2010). This reflects Chinese audiences’ cautious attitude toward foreign cultural content, rooted in the strong emphasis of Chinese culture on collectivism, social harmony, and respect for authority (Fu, 2013). These values, to a certain extent, suppress acceptance of narrative styles that deviate from their cultural norms (Yamato, 2014). Based on these findings, filmmakers targeting markets with similar cultural backgrounds should consider adapting their narrative styles. For example, incorporating elements of teamwork, family values, and other aspects of collectivism (Q. Wang, 2023) can better align content with the audience’s cultural preferences, thereby enhancing audience acceptance and improving the cultural adaptability of films (Brown, 2021).

The MAS dimension’s significant positive impact on viewing motivation suggests that films featuring competitive and achievement-oriented themes resonate strongly with audiences from masculinity-oriented cultures. This finding highlights an opportunity for filmmakers to incorporate competitive or heroism-driven narratives to foster cultural resonance, particularly among the target audiences of American animated films (S.-H. Kim & Lee, 2014). This result aligns with Zhang’s findings, which demonstrate a strong correlation between audiences’ preference for competitive themes and the cultural traits of masculinity (Zhang, 2019). American animated films often emphasize personal struggles and a sense of achievement, which resonate with the values of masculinity-oriented cultures, thereby enhancing viewing motivation. Additionally, VAA and NCI also have significant positive effects on viewing motivation. This indicates that audiences have high expectations for visual quality and narrative sophistication in films (Cutting, 2016; Masuda et al., 2008). For instance, striking visual effects, rich color palettes, and meticulously crafted compositions can significantly enhance audience viewing experiences (Kennedy, 2002). NCI, by engaging cognitive participation and offering re-watch value, has emerged as a key factor in attracting audiences (Busselle & Bilandzic, 2009). Research by Kostoulas et al. shows that narrative complexity transcends cultural boundaries, enhancing a film’s global appeal and emotional resonance (Ros & Kiss, 2018). American animated films excel in both visual and narrative dimensions, which in turn amplifies audience viewing motivation. Preferences for high-quality visual effects and sophisticated storytelling are not merely shaped by cultural backgrounds but are also inherent characteristics of animated films as an art form, capable of capturing audience attention and elevating viewing motivations.

LTO and IVR did not show significant effects on viewing motivation, suggesting that these dimensions may play a secondary role in cross-cultural film acceptance. In particular, the IVR dimension is more closely associated with consumption habits and lifestyle choices rather than directly influencing entertainment content preferences (Heydari et al., 2021). Audiences’ preferences for American animated films may rely more on immediate emotional and visual experiences than on cultural inclinations toward restraint or indulgence (Hofstede & Minkov, 2010). Similarly, as a future-oriented cultural dimension, LTO seems to have limited influence on films as a form of immediate consumption. This indicates that film consumption behavior is driven more by present experiences and short-term emotional factors rather than long-term cultural values (Beugelsdijk & Welzel, 2018). From a practical perspective, filmmakers could address the weaker influence of these cultural dimensions by incorporating more emotionally engaging elements into their films. Such a strategy could attract audiences driven by short-term emotional gratification while enhancing the appeal of the immediate consumption market. This approach would not only compensate for the limited impact of LTO and IVR but also strengthen the connection between audiences and animated films by focusing on instant emotional resonance.

The findings further validate the significant positive relationship between viewing motivation and acceptance, indicating that higher expectations and stronger motivations among audiences lead to higher levels of acceptance. As Igartua and Barrios point out, when viewing motivations are satisfied by a film’s content, audiences experience enhanced emotional engagement and satisfaction (Igartua & Barrios, 2013). Viewing motivation not only influences the choice of films but also shapes overall satisfaction and emotional connection. This underscores the need for filmmakers to delve deeper into audience motivations at the content level, designing emotionally resonant or theme-relevant elements to enhance acceptance (Bartsch, 2012). Moreover, this study highlights the critical role of acceptance in influencing behavioral intentions and brand loyalty. Audiences with high acceptance are more likely to recommend films, engage in repeat viewings, or purchase related merchandise, demonstrating that acceptance fosters positive short-term behaviors and has a long-term impact on brand loyalty (L. Sun et al., 2020). In cross-cultural markets, improving acceptance is particularly important for overcoming cultural barriers and expanding audience bases (Payan & Reardon, 2017). For instance, filmmakers targeting audiences with high preferences for visual aesthetics or narrative complexity can enhance cultural compatibility and emotional appeal by emphasizing these elements. Furthermore, viewing motivation serves as a bridge between acceptance and subsequent behaviors, reflecting the interplay between individual experiences and cultural contexts. This provides filmmakers with data-driven insights for developing content strategies in global markets, allowing for more effective alignment with audience preferences and cultural dynamics.

### 6.2. Theoretical and Practical Implications

The theoretical significance of this study lies in further validating the applicability and explanatory power of Hofstede’s cultural dimensions theory in the context of cross-cultural film acceptance research. Firstly, by incorporating VAA and NCI, this study offers an innovative extension of cultural dimensions theory, addressing its limitations in dynamic media consumption research. Secondly, the findings reveal that combining cultural dimensions with visual and narrative variables provides a more comprehensive explanation of the differentiated impacts on viewing motivation and acceptance, introducing a new perspective that highlights the interaction between culture and individual experiences in cross-cultural communication theories. Lastly, the study further demonstrates the explanatory strength of cultural dimensions theory, particularly within the specific medium of animated films, showcasing its broad applicability in multimedia contexts. This not only offers new research directions for scholars but also underscores the value of a multidimensional perspective in cultural communication and consumption studies.

This study provides clear and actionable recommendations for animated film producers to develop strategies in cross-cultural markets. Firstly, in markets dominated by collectivist cultures (e.g., China), it is advisable to emphasize family values and themes of teamwork to better align with audiences’ cultural expectations. Secondly, for markets with stronger masculinity-oriented cultures, focusing on narratives of personal achievement and competition may be more effective. Additionally, visual aesthetic appeal and narrative complexity are critical factors in enhancing audience acceptance. Producers should prioritize innovation in animation visual styles and narrative design—for instance, incorporating traditional visual elements in collectivist cultures or adopting innovative storytelling techniques in individualistic cultures. Finally, to address cultural differences in global markets, producers can dynamically adjust cultural symbols in film content to broaden the film’s appeal across diverse cultural contexts. The findings also offer insights into cultivating brand loyalty by emphasizing cultural adaptability in the design of film-related merchandise and marketing campaigns, thereby fostering stronger audience brand identification and sustained engagement.

The framework and findings of this study are not only applicable to Chinese audiences but can also be extended to audiences from other cultural backgrounds. For example, in high collectivism cultures like Japan or South Korea, storylines that emphasize teamwork and family values may receive a more positive response. These cultural preferences could influence viewers’ acceptance of film content, particularly in stories that highlight family unity or collective action. In contrast, in individualistic cultures like the United States and Australia, audiences are more likely to appreciate personal heroism and independent, confident character portrayals, making them more receptive to plots featuring protagonists working alone. Similarly, the effects of variables like visual aesthetic appeal and narrative complexity may vary across cultures. In some Western cultures, the visual effects in films may focus more on technological appeal and modern presentations, while in Asian cultures, there may be a greater emphasis on rich colors and intricate visual details. Narrative complexity may be more favored in cultures with low uncertainty avoidance, such as the United States and Europe, where audiences tend to accept complex and open-ended story structures, whereas in high uncertainty avoidance cultures, like China and Japan, viewers may prefer films with clear plots and defined conclusions. Furthermore, future research can apply this framework to audiences in different countries, such as Brazil, India, or Middle Eastern nations, to explore the differences in film acceptance across these cultural contexts. Cross-country comparisons will further validate the impact of cultural dimensions on film reception behavior and assist filmmakers in crafting more culturally adaptive strategies in global markets.

### 6.3. Limitations and Directions for Future Research

This study, based on Hofstede’s cultural dimensions theory, developed a multidimensional research model to analyze the acceptance of American animated films by Chinese audiences. However, the research has the following limitations: Lack of Sample Diversity: The survey participants were predominantly from urban areas, excluding ethnic minorities or subcultural groups in China, which may limit the generalizability and applicability of the findings. Limitations of Data Collection Methods: The self-reported questionnaire format may be subject to social desirability bias and response distortion, potentially affecting the accuracy of the results. Specific Applicability of the Model: While Hofstede’s theory is widely applied, the cultural dimensions’ full relevance to animated films as a specific medium remains to be further validated. For example, elements such as power distance and masculinity may be less pronounced in animation content. Inherent Limitations of Hofstede’s Theory: Although widely adopted, Hofstede’s cultural dimensions face challenges in their applicability and representativeness in the context of contemporary society, characterized by rapid cultural changes and dynamic interactions. Future research should address these limitations by expanding the diversity of samples, adopting mixed methods to minimize biases, and exploring alternative or complementary cultural frameworks to analyze animated film acceptance in cross-cultural contexts.

Future research can explore the following directions. Incorporating Dynamic Cultural Theories: Future studies can integrate theories such as cultural adaptation and cultural hybridity (McSweeney, 2002) to address the static nature of Hofstede’s framework (Fang, 2003), enabling exploration of the dynamic changes in cross-cultural film acceptance behaviors (Kirkman et al., 2006). Introducing Individual-Level Variables: Analyzing individual factors such as age, educational background, and digital literacy could provide more nuanced perspectives on how these variables influence film acceptance, enriching the research on cross-cultural communication. Adapting to Emerging Technologies: With the proliferation of technologies like streaming platforms and VR, future research can investigate how these new media interact with cultural dimensions, such as how Netflix’s globalization strategies adapt to diverse cultural markets. Dynamic Research: With the continuous emergence of new works and technologies, audience preferences and cultural meanings are dynamically evolving. Future research should expand in scope to include the latest and most comprehensive films, providing a more holistic reflection of the evolution of audience acceptance behaviors and the broader cultural significance. For example, emerging media such as streaming platforms and virtual reality technologies introduce new dimensions to audience interaction and engagement with film content, potentially reshaping patterns of film acceptance and the interpretation of cultural values.

## 7. Conclusions

This study integrates Hofstede’s cultural dimensions theory with variables such as visual aesthetic appeal (VAA) and narrative complexity (NCI) to construct a comprehensive analytical framework for exploring the acceptance behaviors and influencing factors of Chinese audiences toward American animated films. The findings reveal that the power distance index (PDI), uncertainty avoidance (UAI), and individualism vs. collectivism (IDV) have a significant negative impact on viewing motivation, with PDI exerting the strongest influence. This suggests that Chinese audiences may prefer film content that aligns with their cultural values, whereas certain themes and narrative styles in American animated films may conflict with the aesthetic preferences of high power distance and collectivist cultural contexts. In contrast, masculinity vs. femininity (MAS), visual aesthetic appeal (VAA), and narrative complexity (NCI) positively influence viewing motivation, indicating that stories emphasizing competition and achievement, along with high-quality visual effects and complex narrative structures, effectively attract audience interest and enhance viewing experiences. Long-term orientation (LTO) and indulgence vs. restraint (IVR) did not exhibit significant negative effects in this study, possibly due to the immediate entertainment nature of animated films, where audiences prioritize short-term emotional engagement over the reflection of long-term cultural values. Additionally, viewing motivation (VM) serves as a bridge by promoting acceptance (AC), acting as the driving force behind audiences’ film choices. When the film content satisfies the audience’s needs, it further enhances their acceptance. Furthermore, acceptance significantly boosts behavioral intentions (BIs), such as recommending the film and repeated viewership, as well as brand loyalty (BL). This indicates that audiences with positive acceptance experiences are more likely to recommend films on social media, re-watch them, and continue supporting related brands, thereby fostering long-term brand loyalty. This study provides new theoretical perspectives and empirical evidence for cross-cultural communication and the film market, highlighting the profound impact of cultural backgrounds on film acceptance behaviors. It not only extends the application of Hofstede’s theory in cultural consumption but also offers practical guidance for filmmakers in designing culturally adaptive strategies within the context of globalization.

Moreover, this study has yielded meaningful findings but is not without limitations, such as the sample scope and the applicability of the theoretical model. Future research could expand sample diversity by including audiences from a broader range of cultural backgrounds and integrating additional cross-cultural theories to analyze film acceptance behaviors from a more dynamic and comprehensive perspective. In conclusion, this study has not only deepened the understanding of Chinese audiences’ behavior in cross-cultural film consumption but also provided theoretical and methodological references for future cross-cultural film research. By further optimizing film content and market strategies, the film industry is well-positioned to achieve broader cultural dissemination and economic benefits in diverse cultural markets.

## Figures and Tables

**Table 1:** Research and discussion on animated films in different aspects.

Aspect	Article	Key Findings
Acceptance of Animated Films	(Cooper-Chen, 2012)	Japanese animation is more popular in Asia than in the West due to regional factors rather than cultural proximity, and overseas exports may save the industry from domestic focus.
	(Westcott, 2011)	The global animation industry has become increasingly challenging due to intense competition in the broadcasting marketplace, with companies ranging from large media conglomerates to small, independent producers.
	(Annett, 2011)	Animation, particularly anime, has fostered transcultural fan communities through asymmetrical, tension-filled interactions between East Asia and the West.
Market Strategies	(Qiu, 2023)	Chinese animation films should prioritize IP marketing and embrace information technology to achieve personalized promotion in the era of ChatGPT.
	(Guo, 2023)	Japanese animation has become a cultural soft power, with streaming media platforms affecting its domestic and international market growth.
Cultural Adaptation	(S.-A. Lee et al., 2017)	Film adaptations of children’s stories from China, South Korea, and Japan use global scripts and techniques to facilitate cross-cultural understanding, overcoming barriers posed by print media.
	(Di Giovanni, 2003)	Disney animated films from the 1990s depict cultural otherness and American values, with the main difficulty in translating the “narrating” (American) culture rather than the “narrated” (remote) culture.
Audience Analysis	(Robinson et al., 2007)	Disney animated films portray most older characters as positive, but a large percentage are portrayed negatively, contributing to children’s negative views of older adults.
	(C. Chen et al., 2022)	Sentiment analysis of animated film reviews using intelligent machine learning can help understand audience emotions and needs, aiding in the promotion of domestic animation films.
Cultural Elements	(Tingting & Fan, 2016)	Chinese elements in animated films, such as Mulan and Kung Fu Panda, show cultural differences and aesthetics, offering a platform for Chinese and Western cultural exchange.
	(Nevin, 2019)	Animation movie costumes play a crucial role in reflecting cultural identity and conveying time and place in the storylines they portray.
Technology and Innovation	(Jiang et al., 2022)	Digital media technology enhances the efficiency and effectiveness of film and television animation design and production, promoting digital development in the industry.
	(Sito, 2013)	Computer graphics, or CG, has revolutionized the art of moving images, leading to multibillion-dollar industries like Toy Story and Avatar.
Legal and Ethical	(Cubitt, 2017)	Ethical approaches to animation differ between hand-drawn and handmade stop-motion, computer-generated imagery, and political simulations, with no clear solution.
	(Slowik, 2013)	Animation’s ability to engage the audience and hold stories still, and second, as it relates to narrative structure and ethical positioning in short films.

**Table 2:** Questionnaire questions.

Variables	Items	Issue	Reference
Power Distance Index (PDI)	PDI1	I believe that the decisions of authority figures in movies are trustworthy.	(Dai et al., 2022) (Begley et al., 2002) (Lei et al., 2021)
	PDI2	I identify with the social hierarchy and power differences portrayed in the movie.	
	PDI3	I support the characters’ obedience to authority figures in the movie.	
	PDI4	I think that the characters’ actions against authority in the movie are justified.	
Uncertainty Avoidance (UAI)	UAI1	I like movie plots that are easy to predict what happens next.	(He, 2020) (S.-A. Lee et al., 2017) (Busselle & Bilandzic, 2009)
	UAI2	I am satisfied with innovative and novel plots in movies.	
	UAI3	I tend to watch movies with clear narratives and simple structures.	
	UAI4	I prefer movies to have a definite ending rather than an open ending.	
Individualism vs. Collectivism (IDV)	IDV1	I appreciate individual heroes in movies who accomplish tasks on their own.	(Warnick et al., 2010) (Cooper-Chen, 2012) (Hernandez & Hirai, 2015)
	IDV2	I enjoy teamwork scenes between characters in movies.	
	IDV3	I identify with the emphasis on family responsibilities in movies.	
	IDV4	I support characters in movies who sacrifice personal interests for the collective good.	
Masculinity vs. Femininity (MAS)	MAS1	I like competitive plots in movies, especially where characters confront each other for success.	(Nave et al., 2020) (Fu & Govindaraju, 2010) (Monleón Oliva, 2020)
	MAS2	I resonate with emotional expressions shown in movies.	
	MAS3	I appreciate characters who pursue personal achievements and success.	
	MAS4	I value interpersonal relationships and interactions among characters in movies.	
Long-Term vs. Short-Term Orientation (LTO)	LTO1	I like plots where characters strive for long-term goals.	(Q. Wang, 2023) (Beugelsdijk & Welzel, 2018) (Hofstede & Minkov, 2010)
	LTO2	I prefer plots where characters achieve immediate success and satisfaction.	
	LTO3	I identify with the portrayal of traditional cultures and values in movies.	
	LTO4	I appreciate characters who emphasize future planning and long-term development.	
Indulgence vs. Restraint (IVR)	IVR1	I enjoy entertaining and humorous plots in movies.	(Nalabandian & Ireland, 2019) (Ishii, 2013) (Crofts, 1992)
	IVR2	I prefer characters who adhere to societal rules and norms.	
	IVR3	I like when characters freely express their emotions and pursue happiness.	
	IVR4	I admire characters in movies who demonstrate self-control and overcome impulses.	
Visual Aesthetic Appeal (VAA)	VAA1	I am satisfied with the use of colors in movies.	(Brown, 2021) (Jiang et al., 2022) (Kostoulas et al., 2017)
	VAA2	I think the animation effects in movies are outstanding.	
	VAA3	I enjoy the unique visual styles and artistic expressions in movies.	
	VAA4	I am satisfied with the appearance design of characters in movies.	
Narrative Complexity (NCI)	NCI1	I like complex and multi-layered narrative structures in movies.	(Cutting, 2016) (X. Chen, 2018) (Busselle & Bilandzic, 2009)
	NCI2	I appreciate the growth and development of characters in movies.	
	NCI3	I am satisfied with novel or unique narrative styles in movies.	
	NCI4	I like movies with a moderate pace of plot development, neither too fast nor too slow.	
Viewing Motivation (VM)	VM1	I watch movies mainly for entertainment and relaxation.	(Bartsch, 2012; Rubin, 1981) (Du et al., 2022)
	VM2	I watch movies to experience emotional resonance or emotional release.	
	VM3	I watch movies because I am interested in the movie’s theme or genre.	
	VM4	I watch movies to share the experience with family or friends.	
Acceptance (AC)	AC1	The movie met my overall expectations and preferences.	(X. Lu & Lo, 2007) (Bartsch, 2012) (Picón-Berjoyo et al., 2016)
	AC2	Watching the movie provided me with an enjoyable experience.	
	AC3	I am satisfied with the storyline, characters, and visual presentation of the movie.	
	AC4	I find the movie appealing and worth recommending.	
Behavioral Intentions (BIs)	BI1	If I like this movie, I will recommend it to my friends and family.	(Shieh & Lin, 2022) (Picón-Berjoyo et al., 2016) (Danaher & Dagger, 2012)
	BI2	If this movie leaves a good impression on me, I will buy its DVD or other related merchandise.	
	BI3	I am willing to post positive reviews on social media if I am satisfied with this movie.	
	BI4	If I rate this movie highly, I might watch it again.	
Brand Loyalty (BL)	BL1	I tend to follow and support the film companies that produce movies I like.	(J. Kim et al., 2008) (Jacoby & Kyner, 1973) (Picón-Berjoyo et al., 2016)
	BL2	I am inclined to rewatch my favorite movie series.	
	BL3	I usually have high expectations for new works from my favorite movie production brands.	
	BL4	If a movie meets my expectations, I will continue to follow and support that brand.	

**Table 3:** Selected films.

Film	Year	Box Office (Billion USD)	Budget (Billion USD)	Duration
The Lion King	1994	9.82	0.45	88 min
Zootopia	2016	10.25	O	108 min
Inside Out	2015	8.59	1.75	95 min
Spider-Man: Into the Spider-Verse,	2018	3.94	0.90	116 min
The Incredibles,	2004	6.32	0.92	115 min
How to Train Your Dragon,	2010	4.95	1.65	98 min
Frozen,	2013	13.06	1.50	102 min
Toy Story	1995	3.94	O	81 min
Finding Nemo,	2003	9.42	0.94	100 min
Madagascar 3: Europe’s Most Wanted	2012	7.47	1.45	93 min
Monsters, Inc.	2001	5.80	1.15	92 min
COCO	2017	8.15	O	105 min
Despicable Me 3	2017	10.34	0.80	96 min
Monsters University	2013	7.43	O	110 min
Ice Age	2002	3.83	0.59	81 min

**Table 4:** Descriptive statistics of the basic information of the participants.

Item	Indicators	Frequency	Percent (%)
Gender	Male	247	48.7
	Female	260	51.3
Age	Under 18	79	15.6
	18–25	187	36.9
	26–30	127	25
	31–40	86	17
	41–50	28	5.5
Academic Qualifications	High school and below	91	17.9
	College/Undergraduate	374	73.8
	Postgraduate and above	42	8.3

**Table 5:** Statistics on animated film viewing habits.

Item	Response		Popularity Rate %
	*n*	Response Rate %	
The Lion King (1994)	429	8.311	84.615
Zootopia (2016)	448	8.679	88.363
Inside Out (2015)	172	3.332	33.925
Spider-Man: Into the Spider-Verse (2018)	240	4.649	47.337
The Incredibles (2004)	253	4.901	49.901
How to Train Your Dragon (2010)	215	4.165	42.406
Frozen (2013)	393	7.613	77.515
Toy Story (1995)	427	8.272	84.221
Finding Nemo (2003)	457	8.853	90.138
Monsters, Inc. (2001)	167	3.235	32.939
Coco (2017)	202	3.913	39.842
Despicable Me (2017)	454	8.795	89.546
Ice Age (2009)	447	8.659	88.166
Monsters University (2013)	448	8.679	88.363
Madagascar (2012)	410	7.943	80.868
Total	5162	100.00	1018.146

**Table 6:** Common method bias test.

Component	Initial Eigenvalues		Extraction Sums of Squared Loadings			
	Total	% of Variance	Cumulative %	Total	% of Variance	Cumulative %
1	15.480	32.249	32.249	15.480	32.249	32.249
2	2.160	4.501	36.750	2.160	4.501	36.750
3	2.077	4.326	41.076	2.077	4.326	41.076
4	1.972	4.109	45.185	1.972	4.109	45.185
5	1.886	3.929	49.114	1.886	3.929	49.114
6	1.841	3.836	52.950	1.841	3.836	52.950
7	1.682	3.505	56.455	1.682	3.505	56.455
8	1.617	3.369	59.824	1.617	3.369	59.824
9	1.590	3.313	63.137	1.590	3.313	63.137
10	1.503	3.132	66.269	1.503	3.132	66.269
11	1.457	3.035	69.304	1.457	3.035	69.304
12	1.378	2.872	72.176	1.378	2.872	72.176

**Table 7:** Reliability test.

Variables		CITC	CA	
PDI	PDI1	0.693	0.824	0.858
	PDI2	0.694	0.824	
	PDI3	0.702	0.820	
	PDI4	0.723	0.811	
UAI	UAI1	0.707	0.820	0.859
	UAI2	0.700	0.823	
	UAI3	0.704	0.821	
	UAI4	0.708	0.820	
IDV	IDV1	0.671	0.841	0.863
	IDV2	0.724	0.820	
	IDV3	0.721	0.821	
	IDV4	0.728	0.818	
MAS	MAS1	0.739	0.841	0.877
	MAS2	0.736	0.842	
	MAS3	0.761	0.832	
	MAS4	0.704	0.854	
LTO	LTO1	0.726	0.825	0.866
	LTO2	0.702	0.835	
	LTO3	0.723	0.826	
	LTO4	0.713	0.830	
IVR	IVR1	0.716	0.829	0.866
	IVR2	0.722	0.826	
	IVR3	0.716	0.829	
	IVR4	0.710	0.831	
VAA	VAA1	0.749	0.840	0.878
	VAA2	0.735	0.845	
	VAA3	0.740	0.843	
	VAA4	0.725	0.849	
NCI	NCI1	0.719	0.810	0.857
	NCI2	0.692	0.821	
	NCI3	0.694	0.820	
	NCI4	0.696	0.819	
VM	VM1	0.714	0.820	0.861
	VM2	0.693	0.829	
	VM3	0.706	0.824	
	VM4	0.717	0.819	
AC	AC1	0.745	0.842	0.879
	AC2	0.737	0.845	
	AC3	0.727	0.849	
	AC4	0.744	0.842	
BI	BI1	0.714	0.828	0.865
	BI2	0.717	0.827	
	BI3	0.695	0.836	
	BI4	0.731	0.821	
BL	BL1	0.707	0.826	0.862
	BL2	0.720	0.820	
	BL3	0.706	0.826	
	BL4	0.705	0.827	

Note: CITC = Corrected Item–Total Correlation.

**Table 8:** KMO and Bartlett’s test.

Kaiser–Meyer–Olkin test		0.944
Bartlett’s Test of Sphericity	Approx. Chi-Square	13,861.344
	df	1128.000
	Sig.	0.000

**Table 9:** Confirmatory factor model fit.

	Results of This Study	Reference Range
χ^2^/df	1.046	“When the sample size is less than 300, <3; when the sample size is between 300 and 1000, <7; when the sample size exceeds 1000, it is not considered.”
RMSEA	0.01	<0.05: Excellent; <0.08: Good
SRMR	0.036	<0.05: Excellent; <0.08: Good
NFI	0.926	>0.9: Excellent; >0.8: Acceptable
CFI	0.996	>0.9
GFI	0.921	>0.9: Excellent; >0.8: Acceptable

**Table 10:** Correlation analysis and discriminant validity test.

	PDI	UAI	IDV	MAS	LTO	IVR	VAA	NCI	VM	AC	BI	BL
PDI	0.776											
UAI	0.542	0.778										
IDV	0.428	0.542	0.783									
MAS	−0.478	0.470	0.428	0.801								
LTO	0.498	−0.476	0.470	−0.478	0.786							
IVR	0.452	0.464	−0.508	−0.476	0.498	0.786						
VAA	−0.514	0.514	0.535	−0.508	0.464	0.452	0.802					
NCI	−0.432	−0.523	0.411	−0.518	0.535	0.514	−0.514	0.774				
VM	−0.512	−0.470	−0.435	−0.452	−0.518	0.411	−0.523	−0.432	0.780			
AC	−0.445	−0.524	−0.425	0.463	0.499	−0.452	−0.435	−0.470	−0.512	0.803		
BI	−0.435	−0.479	−0.479	0.471	−0.509	0.499	0.463	−0.425	−0.524	−0.445	0.785	
BL	−0.420	−0.458	−0.458	0.505	−0.504	−0.495	−0.509	0.471	−0.479	−0.479	−0.435	0.781

**Table 11:** Fit index interpretation and reference values for evaluation.

Parameter Name	Explanation and Reference for Numerical Evaluation
CMIN/DF (χ^2^/df)	CMIN/DF represents the chi-square/degrees of freedom ratio. Generally, a value less than 3.0 indicates good model fit. When the value is between 3 and 5, the fit is acceptable.
GFI	GFI is the Goodness of Fit Index. A value between 0.85 and 0.9 is acceptable, and a value above 0.9 is considered good.
AGFI	AGFI is the Adjusted Goodness of Fit Index, with values ranging between 0 and 1. The closer the value is to 1, the better the model fit. A value between 0.8 and 0.9 is acceptable, and a value above 0.9 is considered good.
NFI, TLI, CFI, IFI	CFI (Comparative Fit Index), NFI (Normed Fit Index), and TLI (Tucker–Lewis Coefficient): The closer the values of these indices are to 1, the better the model fit. Generally, values close to 0.9 are required, and values ≥ 0.95 indicate a perfect model fit.
RMSEA	RMSEA is the Root Mean Square Error of Approximation. A lower value indicates better model fit. A value below 0.05 represents a good fit, and a value below 0.08 is considered acceptable.

**Table 12:** Model fit.

Model Evaluation Indicators	Fit Criteria		Values for This Study
	Good	Acceptable	
χ^2^	Preferably Low	Preferably Low	1383.481
χ^2^/df	1–3	3–5	1.329
GFI	>0.90	>0.85	0.903
AGFI	>0.90	>0.80	0.89
RMSEA	<0.05	<0.08	0.025
NFI	>0.9	>0.85	0.904
CFI	>0.9	>0.85	0.974

**Table 13:** Significance test of path coefficients.

Path Relationship			Estimate	Standardized Estimate	S.E.	C.R.	*p*
VM	<--	PDI	−0.167	−0.157	0.060	−2.783	0.005
VM	<--	UAI	−0.161	−0.160	0.059	−2.729	0.006
VM	<--	IDV	−0.153	−0.138	0.060	−2.541	0.011
VM	<--	MAS	0.143	0.146	0.054	2.650	0.008
VM	<--	LTO	−0.018	−0.019	0.056	−0.324	0.746
VM	<--	IVR	−0.037	−0.038	0.053	−0.698	0.485
VM	<--	VAA	0.122	0.134	0.050	2.420	0.016
VM	<--	NCI	0.160	0.160	0.054	2.977	0.003
AC	<--	VM	0.606	0.567	0.055	10.963	0.000
BI	<--	AC	0.371	0.405	0.047	7.895	0.000
BL	<--	AC	0.414	0.454	0.047	8.802	0.000

## Data Availability

All data generated or analyzed during this study are included in this article. The raw data are available from the corresponding author upon reasonable request.
